# Acute myeloid leukemia stem cell markers in prognosis and targeted therapy: potential impact of BMI-1, TIM-3 and CLL-1

**DOI:** 10.18632/oncotarget.11063

**Published:** 2016-08-05

**Authors:** Noureldien H.E. Darwish, Thangirala Sudha, Kavitha Godugu, Osama Elbaz, Hasan A. Abdelghaffar, Emad E.A. Hassan, Shaker A. Mousa

**Affiliations:** ^1^ Faculty of Medicine, Mansoura University, Mansoura, Egypt; ^2^ The Pharmaceutical Research Institute, Albany College of Pharmacy and Health Sciences, Rensselaer, NY, USA

**Keywords:** acute myeloid leukemia, leukemic stem cells, chronic myeloid leukemia, chronic myelomonocytic leukemia, PTC-209

## Abstract

Acute myeloid leukemia (AML) patients show high relapse rates and some develop conventional chemotherapy resistance. Leukemia Stem Cells (LSCs) are the main player for AML relapses and drug resistance. LSCs might rely on the B-cell-specific Moloney murine leukemia virus integration site-1 (*BMI-1*) in promoting cellular proliferation and survival. Growth of LSCs in microenvironments that are deprived of nutrients leads to up-regulation of the signaling pathways during the progression of the disease, which may illustrate the sensitivity of LSCs to inhibitors of those signaling pathways as compared to normal cells. We analyzed the expression of LSC markers (CD34, CLL-1, TIM-3 and BMI-1) using quantitative RT-PCR in bone marrow samples of 40 AML patients of different FAB types (M1, M2, M3, M4, M5, and M7). We also studied the expression of these markers in 2 AML cell lines (Kasumi-1 and KG-1a) using flow cytometry and quantitative RT-PCR. The overexpression of TIM-3, CLL-1, and BMI-1 was markedly correlated with poor prognosis in these patients. Our *in vitro* findings demonstrate that targeting BMI-1, which markedly increased in the leukemic cells, was associated with marked decrease in leukemic burden. This study also presents results for blocking LSCs' surface markers CD44, CLL-1, and TIM-3. These markers may play an important role in elimination of AML. Our study indicates a correlation between the expression of markers TIM-3, CLL-1, and especially of BMI-1 and the aggressiveness of AML and thus the potential impact of prognosis and therapies that target LSCs on improving the cure rates.

## INTRODUCTION

Acute myeloid leukemia (AML) is a hematological disease characterized by specific clinical and molecular heterogeneous disorders. AML is associated with poor long-term survival, even with newer chemotherapeutic agents. Various studies reported that AML relapse and resistance to chemotherapies may originate from a small clone, known as Leukemic Stem Cells (LSCs) [1]. LSCs are identified as a chemo-resistant clone that has the ability for limitless self-renewal and also production of a large number of blast cells [2]. The identification and targeting of LSCs is the hope for improvement of long-term survival for AML patients [3]. Therefore, it is necessary to identify signaling pathways and surface and molecular markers that are specific to the LSCs in order to eliminate them without any damage to the normal hematopoietic stem cells (HSCs) [3].

It was reported that LSCs were present within the CD34+/CD38- clone because of the ability to establish human AML in a xenograft model [4]. Several surface markers reported to be expressed on the LSCs' surface are antigens such as CD33 [5], CD123 [6], CLL-1 [7], CD44 [8], CD47 [9], and recently TIM-3 [10]. On the other hand, the B-cell-specific Moloney murine leukemia virus integration site-1 (*BMI-1* gene) is a member of the polycomb group genes family [11]. The polycomb group gene *BMI-1* participates in the growth and regulation of cell proliferation of both normal and LSCs [12].

In 2010, there were more than 12,000 new cases of AML in the United States (0.8% of all new cancer cases and 29% of all leukemia cases) [13], and early death (within two months after diagnosis) was observed in 52.7% of older AML patients [14]. There is no recent data about the incidence of all leukemia in Egypt, but the U.S. National Cancer Institute (NCI) has reported that between 1999 and 2001 the incidence of all leukemia in Egypt was 6.0 per 100,000, while the incidence of AML was 1.1 per 100,000 [15]. Although over the last few decades there has been some improvement in survival for AML patients, mainly in younger age groups, AML long-term survival is still a big challenge [13].

This study was designed to investigate markers such as CD34, CD44, CLL-1, TIM-3 and BMI-1 expression in samples from myeloid leukemia patients and whether they might serve as biomarkers to predict disease aggressiveness. We also studied the effect of targeting these markers on the leukemic clone proliferation and survival.

## RESULTS

### Patients' assessment for LSCs markers

**Figure 1:**
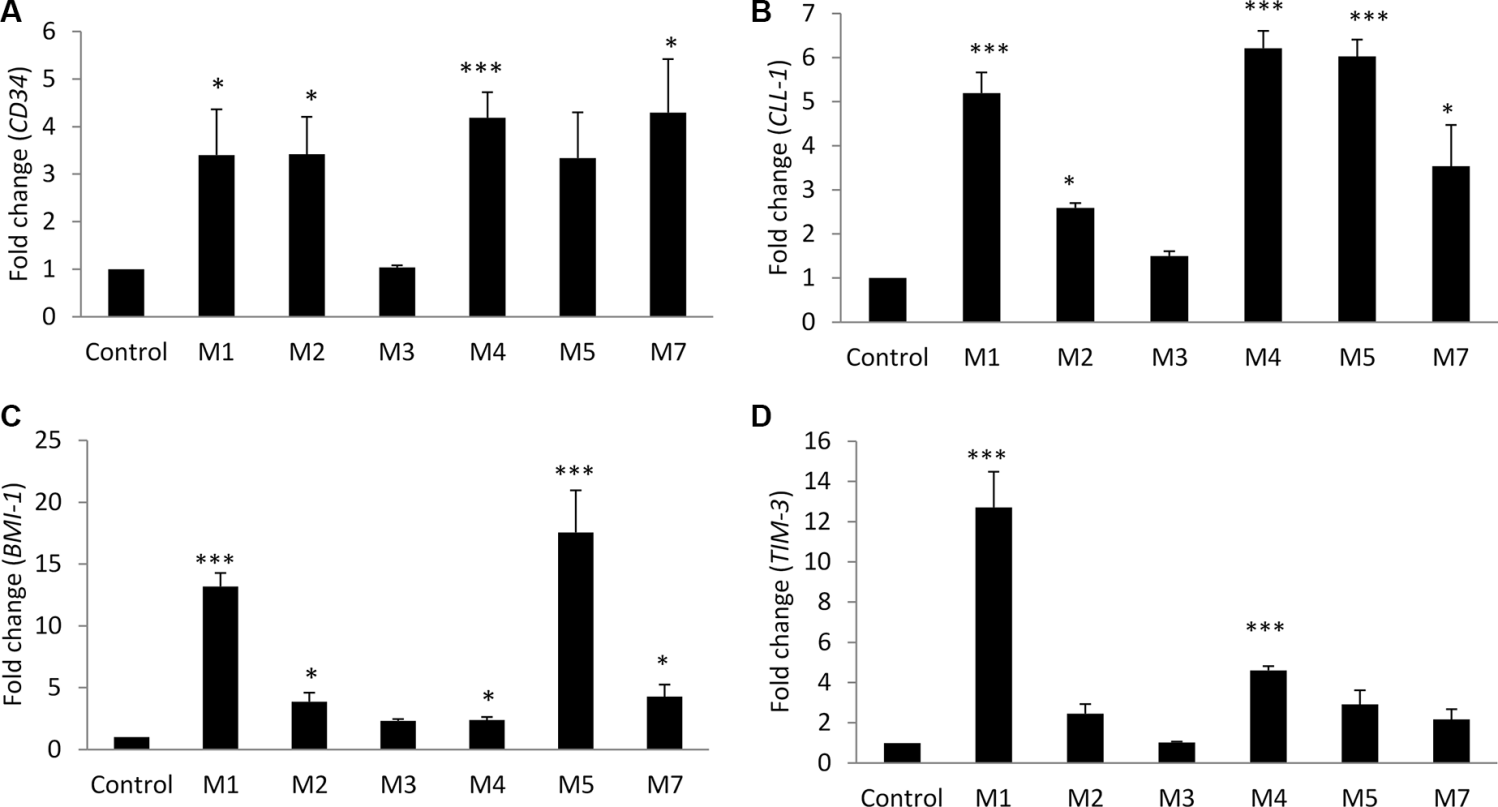
Leukemic stem cell genes expression analysis by qRT-PCR in bone marrow samples from AML patients versus control subjects Gene expression of: (**A**) *CD34*, (**B**) *CLL-1*, (**C**) *BMI-1*, and (**D**) *TIM-3*. Fold changes in the respective gene expression are expressed as mean ± S.E.M., *n* = 10 control and 40 patients (M1 = 5, M2 = 7, M3 = 5, M4 = 16, M5 = 4, M7 = 3). One-way ANOVA was used followed by the Newman-Keuls post-test (***p < 0.001, *p < 0.05).

Gene expression analysis was done with qRT-PCR technique. We found expression of *CD34*, *CLL-1*, *BMI-1* and *TIM-3* was increased in all AML groups except M3. *CD34* expression increased in comparison to the control group (3.4–4.3 fold), with no specific correlation to any French-American-British (FAB) classification (Figure 1A). *CLL-1* showed high expression in M1, M4, M5, and M7 groups (3.5–6 fold), while the expression in M2 group was less (2.5 fold) compared to the control group (Figure 1B).

*BMI-1* was found to be highly expressed compared to control in M1 (13.2 fold), M5 (17.5 fold), and to a lesser extent in M7 (4.3 fold), and only 2.3–3.5 fold increase in M2, M3, and M4 groups (Figure 1C). The high expression of *BMI-1* in M1, M5, and M7 groups was confirmed by Western blot technique (data not shown).

The expression of TIM-3 showed marked statistical significance in M1 and M4 groups (12.7 and 4.6 fold, respectively), and less in M2, M5 and M7 groups (2.2–2.9 fold) (Figure 1D).

**Figure 2:**
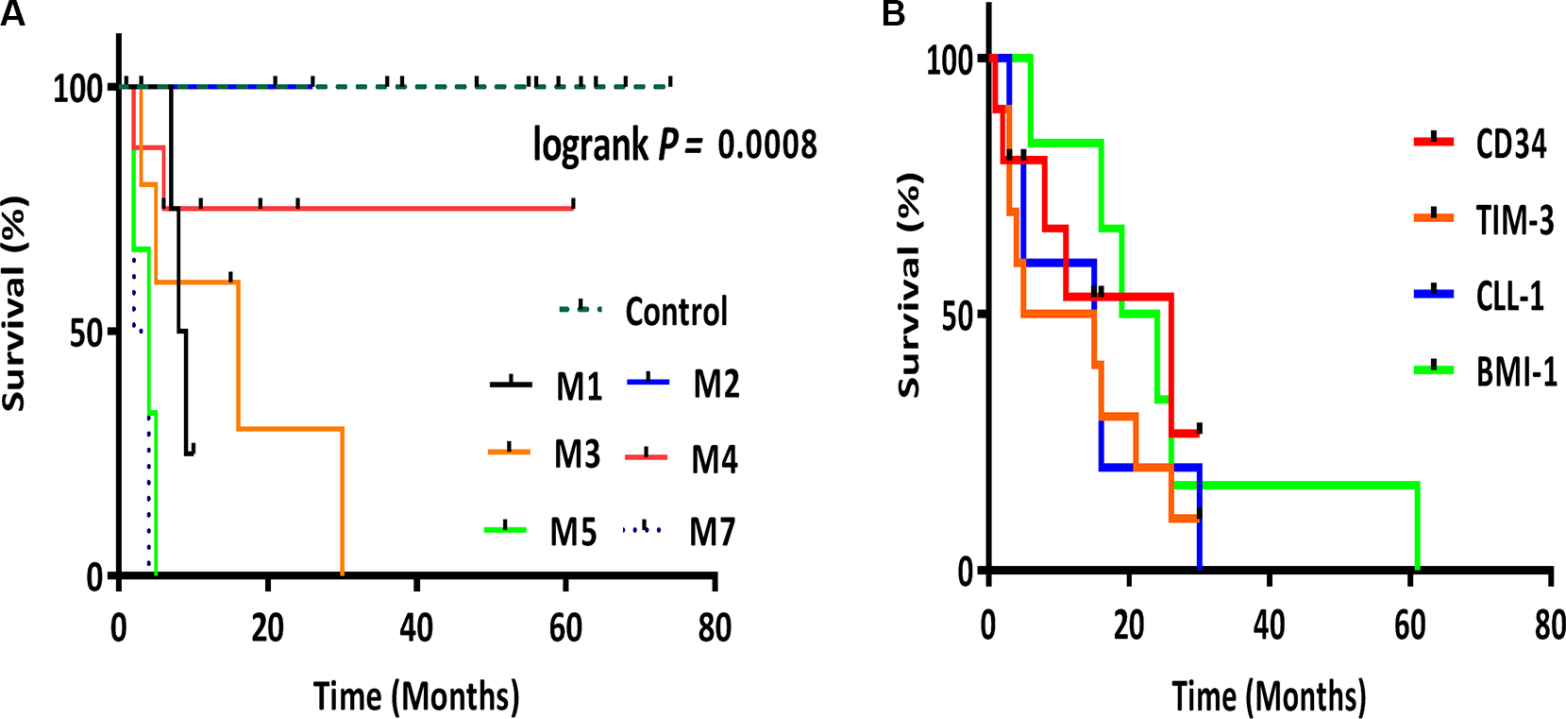
Kaplan-Meier survival analysis (**A**) Kaplan-Meier plot representing the overall survival for AML groups M1, M2, M3, M4, M5 and M7. (**B**) Kaplan-Meier plot representing the correlation between LSC genes *CD34*, *TIM-3*, *CLL-1* and *BMI-1* expression and survival. *CD34* (*p* = 0.0022), *TIM-3* (*p* = 0.0035), *CLL-1* (*p* = 0.0006), *BMI-1* (*p* < 0.001). Patients with low gene expression had longer survival time than those with high expression. For Kaplan-Meier plots, the log-rank test was applied. The log-rank test *p* value indicates the significance of the correlation.

For patient outcomes, the LSCs markers, especially BMI-1, were significantly high in 12 patients (M1, M5, and M7); 8 died, 2 relapsed, 1 did not come back to follow up, and only 1 patient showed complete remission. Twenty-eight patients (M2, M3, and M4) associated with mild to moderate increase in expression of LSC markers; 6 died, 4 relapsed, 8 did not come back to follow up, 1 showed resistance to chemotherapy, and 9 had complete remission. The overall survival for our AML patients indicates that patients with low gene expression had longer survival time than those with high expression (Figure 2). BMI-1 and CLL-1 expressions were significantly increased in patients with AML FAB M5 (17 fold), which was associated with the worst prognosis. Patients with AML FAB M4 and M2 were associated with mild increase (2.7 and 3.5 fold, respectively), which was associated with better prognosis.

### *In vitro* study

The flow cytometry stem cell marker assay for the Kasumi-1 and KG-1a cell lines was performed to study the expression of CD34, CD38, CD44, TIM-3, CLL-1 and BMI-1 (Supplementary Data, Table S1). Kasumi-1 cell line showed a positive shift for CD34 (63%) and CD38 (4.1%), with a double expression (1.6%). TIM-3 expression was 16% and had double expression with CD34 (9.3%). CLL-1 expression was 8.4% and had double expression with CD34 (4.2 %) (Supplementary Data, Figure S1). For the KG-1a cell line, almost all cells expressed CD34 (98%) and CD38 (26%), with a double expression (25%). TIM-3 expression was (1.3%), with double expression with CD34 (1.2 %). CLL-1 expression was 4%, and double expression with CD34 was about 3.7 % (Supplementary Data, Figure S2). Expression of CD44 and BMI-1 in both cell lines was more than 90%. About 61% of Kasumi-1 cells had double expression of CD34 and CD44, while almost all CD44 positive KG-1a cells also expressed CD34.

### Cell vitality assay

We used the same AML cell lines to assay the effect of targeting of certain surface molecules (with monoclonal antibodies) and intracellular molecules (with small molecule inhibitors) on the cell proliferation and also to find the optimal concentration. Assessment for cell proliferation before running MTT assay was important to determine the appropriate cell count and duplication time for each cell line. The appropriate cell count for both cell lines was 50–100 × 10^6^/ well. Kasumi-1 cells' duplication time was about 72 hours while KG-1a was 48 hours. We used different concentrations to determine the appropriate concentration that would interfere with cell proliferation.

**Figure 3:**
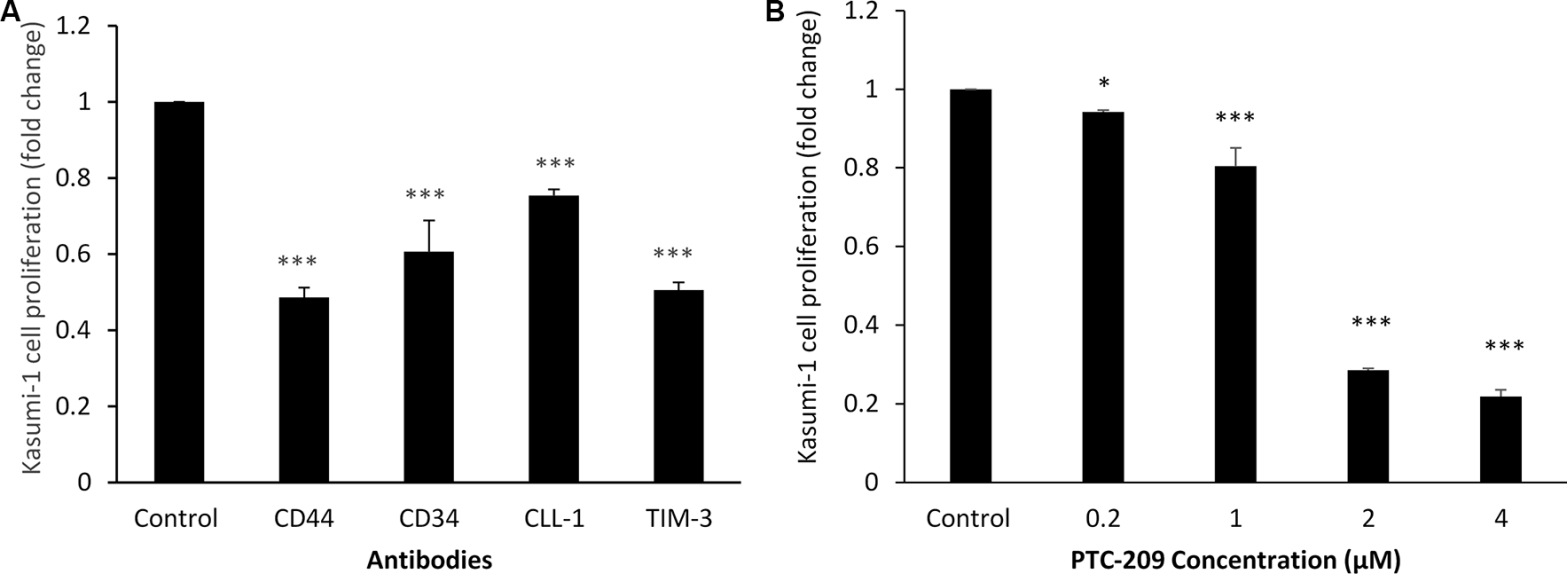
Kasumi-1 cell line proliferation with antibodies against CD44, CD34, CLL-1, TIM-3 or BMI-1 small molecular inhibitor PTC-209, measured with MTT assay (**A**) Anti-CD44 (1.0 μg/100 μl), anti-CD34 (1.0 μg/100 μl), anti-CLL-1 (0.5 μg/100 μl), and anti-TIM-3 (0.5 μg/100 μl). (**B**) PTC-209 at concentrations ranging from 0.2 to 4 μM. Fold changes in cell proliferation are expressed as mean ± S.D., *n* = 3. One-way ANOVA was used followed by the Newman-Keuls post-test (***p < 0.001, **p < 0.05).

For Kasumi-1 cells the 72 hour assay showed that the lowest concentration of anti-CD44 (1 μg/100 μl) was associated with a 50% decrease in proliferation (*p* < 0.001), anti-CD34 (1 μg/100 μl) with a decrease of about 40% (*p* < 0.001), and anti-CLL-1 (1, 0.5, 0.05 μg/100 μl) with about 24% decrease, all compared to control. The best result was obtained with anti-TIM-3 (0.5 μg/100 μl), which decreased proliferation by about 49% (*p* < 0.001) compared to control (Figure 3A). BMI-1 small molecule inhibitor PTC-209 at 2 μM was found to be the best concentration; it decreased proliferation by about 70% (*p* < 0.001) compared to control (Figure 3B).

**Figure 4:**
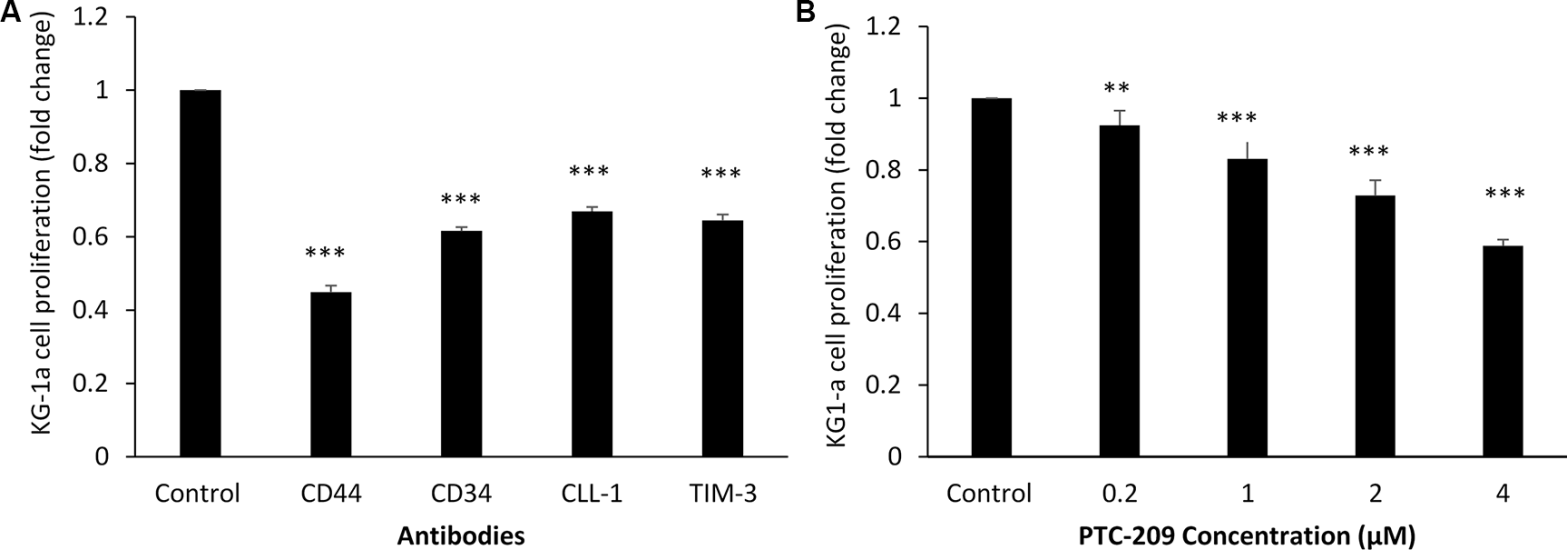
KG-1a cell line proliferation with antibodies against CD44, CD34, CLL-1, TIM-3 or BMI-1 small molecular inhibitor PTC-209, measured with MTT assay (**A**) Anti-CD44 (1.0 μg/100 μl), anti-CD34 (1.0 μg/100 μl), anti-CLL-1 (0.5 μg/100 μl), and anti-TIM-3 (0.5 μg/100 μl). (**B**) PTC-209 at concentrations ranging from 0.2 to 4 μM. Fold changes in cell proliferation are expressed as mean ± S.D., *n* = 3. One-way ANOVA was used followed by the Newman-Keuls post-test (***p < 0.001, **p < 0.05).

For the KG-1a cell line, the 48 hour assay showed that the lowest concentration of anti-CD44 and anti-CD34 (1 μg/100 μl) decreased cell proliferation by about 55% and 39%, respectively, compared to control (*p* < 0.001). Anti-CLL-1 (0.5 μg/100 μl) was associated with a smaller decrease in proliferation (30%). The best result was obtained with anti-TIM-3 (0.5 μg/100 μl), which decreased proliferation by about 35% compared to control (*p* < 0.001) (Figure 4A). The BMI-1 small molecule inhibitor PTC-209 at 4 μM decreased cell proliferation by 41% compared to control (*p* < 0.001), and at 2 μM it decreased the cell proliferation by 28% (*p* < 0.001) (Figure 4B).

**Figure 5:**
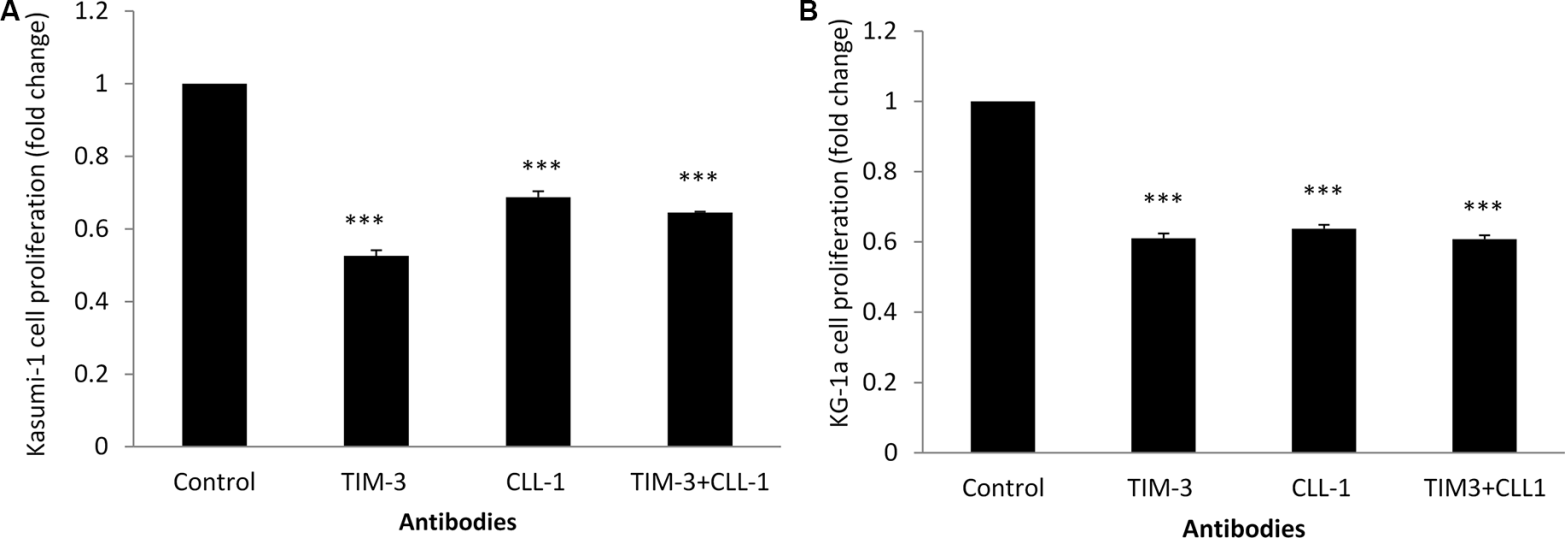
Kasumi-1 and KG-1a cell lines proliferation with antibodies against CLL-1 and TIM-3 measured with MTT assay (**A**) Kasumi-1 cell line with combined anti-CLL-1 (0.5 μg/100 μl) and anti-TIM-3 (0.5 μg/100 μl). (**B**) KG-1a cell line with combined anti-CLL-1 (0.5 μg/100 μl) and anti-TIM-3 (0.5 μg/100 μl). Fold changes in cell proliferation are expressed as mean ± S.D., *n* = 3. One-way ANOVA was used followed by the Newman-Keuls post-test (***p < 0.001, **p < 0.05).

Association of anti-CLL-1 and anti-TIM-3 didn't show any synergism effect in either Kasumi-1 (Figure 5A) or KG-1a cell lines (Figure 5B).

## DISCUSSION

For this study of the gene expression of both well-known and lesser known LSC molecules, we preferred to use bone marrow samples because they are more representative and richer in stem cells in comparison to peripheral blood. We chose to use Kasumi-1 and KG-1a cell lines because these cells do not spontaneously differentiate to granulocyte and eosinophilic-like cells and do not respond to colony stimulating factor (Supplementary Data, Figure S3).

### Known LSCs markers

### CD34

CD34, a stem cell marker, is expressed by blast cells only for some AML patients. It is controversial as to whether CD34 expression (defined as at least 10–20% positive cells) has any prognostic value in AML patients who receive intensive chemotherapy [16]. Some previous studies suggested that CD34 expression is always associated with multidrug resistance P-glycoprotein overexpression, which may explain the poor response to treatment. However, the relation between the overexpression of multidrug resistance P-glycoprotein and CD34, as well as the relation between the expression of multidrug resistance P-glycoprotein and the poor prognosis, needs further assessment [17].

Our present results evaluating the expression and prognostic effect of CD34 were conflicting because there was heterogeneity in gene expression profiles among AML patients. CD34 increased in almost all patients with no specific correlation to a certain FAB group. The most commonly used systems to classify AML into subtypes is the French-American-British (FAB) classification and the newer World Health Organization (WHO) classification. The FAB system is useful and still widely used in the clinical field, especially in our country (Egypt) [18]. Only the M3 group showed CD34 expression close to the control group. Oyan *et al.* reported the same results when they tried to find the relation between CD34 expression and prognosis in AML patients [16]. We suggest that CD34 may play an important role in cell survival and maturation because CD34 expressed mainly on progenitors cells and disappeared with cell maturation. Our data showed a marked decrease in Kasumi-1 and KG-1a cell proliferations even at the lowest concentrations of anti-CD34, which is consistent with the role of CD34 in cell survival and the differentiation process.

### CD44

CD44 is known to play an important role in hematopoiesis including cell migration, proliferation, differentiation, and survival of hematopoietic stem/progenitor cells. LSCs were known to be associated with CD44 overexpression in comparison to normal HSCs [19]. Our work shows the high expression of CD44 on both cell lines with the dramatic effect of blocking mAbs, suggesting the multi-functionality of CD44 not only on homing and engraftment but also in cell proliferation and signaling for cell survival. Consistent with our findings, Hertweck *et al.* reported that CD44, through complex intracellular signaling cascades including the PI3K/Akt and the Ras/ERK pathway, mediates CD44-regulated adhesion, migration, proliferation, survival, and apoptosis [20]. Jin *et al.* reported the important role of CD44 in homing and engraftment of LSCs in the bone marrow niche [8]. They used H90 mAbs to demonstrate the effect of targeting of CD44 on homing and engraftment of leukemic cells in bone marrow. Lapidot and colleagues showed that crosstalk exists between the CXCR4–SDF-1 axis and CD44 in normal hematopoietic cells and also that the use of specific antibodies disrupting those interactions blocked AML cell homing [21].

### CLL-1

CLL-1 is a type II transmembrane glycoprotein and member of the large family of C-type lectin-like receptors involved in immune regulation [22]. It is a unique markers for LSCs, expressed in more than 87% of AML patients, with weak expression in normal hematopoietic cells [23]. For AML, both blast cells and LSCs express CLL-1 (86.5% vs. 54.5%, respectively) [22]. CLL-1 intracellular domain consists of an immunotyrosine-based inhibition motif and an YXXM motif, suggesting a role for CLL-1 as a signaling receptor. The immunotyrosine-based inhibition motif containing receptors works as an inhibitor for the active pathways via recruitment of the protein tyrosine phosphatases SHIP, SHP-1, and SHP-2 [24]. On the other hand, the YXXM motif carries a potential p85 subunit binding site (SH2 domain) of the phosphatidylinositol 3′ kinase, which has an important role in cellular activation pathways [25]. The signal function of CLL-1 depends on whether its ligand leads to activation of YXXM motif or immunotyrosine-based inhibition motif, which may depend on the level of phosphorylation of both motifs as well as on the efficiency of recruitment of SHP-1/2 and p85. Besides its role as a cellular activation motif, the YXXM motif has been demonstrated to act as an internalization motif [26, 27]. It might be responsible for the demonstrated internalization of the CLL-1 receptor upon antibody-mediated cross-linking. Furthermore, the CLL-1 internalization upon ligand encounter may instead act as a switch to terminate receptor-mediated signaling.

Bakker *et al.* described the pattern of expression of CLL-1 in hematopoietic cells and found that the expression of CLL-1 was in myeloid cells, as well as in the majority of AML blasts [28]. A recent study indicated that CLL-1 is also present on the majority of the CD34^+^/CD38^−^ compartment in AML but absent from CD34^+^/CD38^−^ cells in normal and in regenerating bone marrow controls, which has an important role in discrimination between leukemic and normal stem cells [7]. On the other hand, anti-CLL-1 antibody was unlikely to have an anti-leukemic effect unlike the other mAbs because it had only a very mild effect on cell proliferation in both cell lines as found with our data. Consistent with our findings, work by van Rhenen and colleagues showed that anti-CLL-1 mAbs did not influence engraftment of AML CD34+ cells in NOD/SCID mice [7].

### Potential new markers

### TIM-3

**Figure 6:**
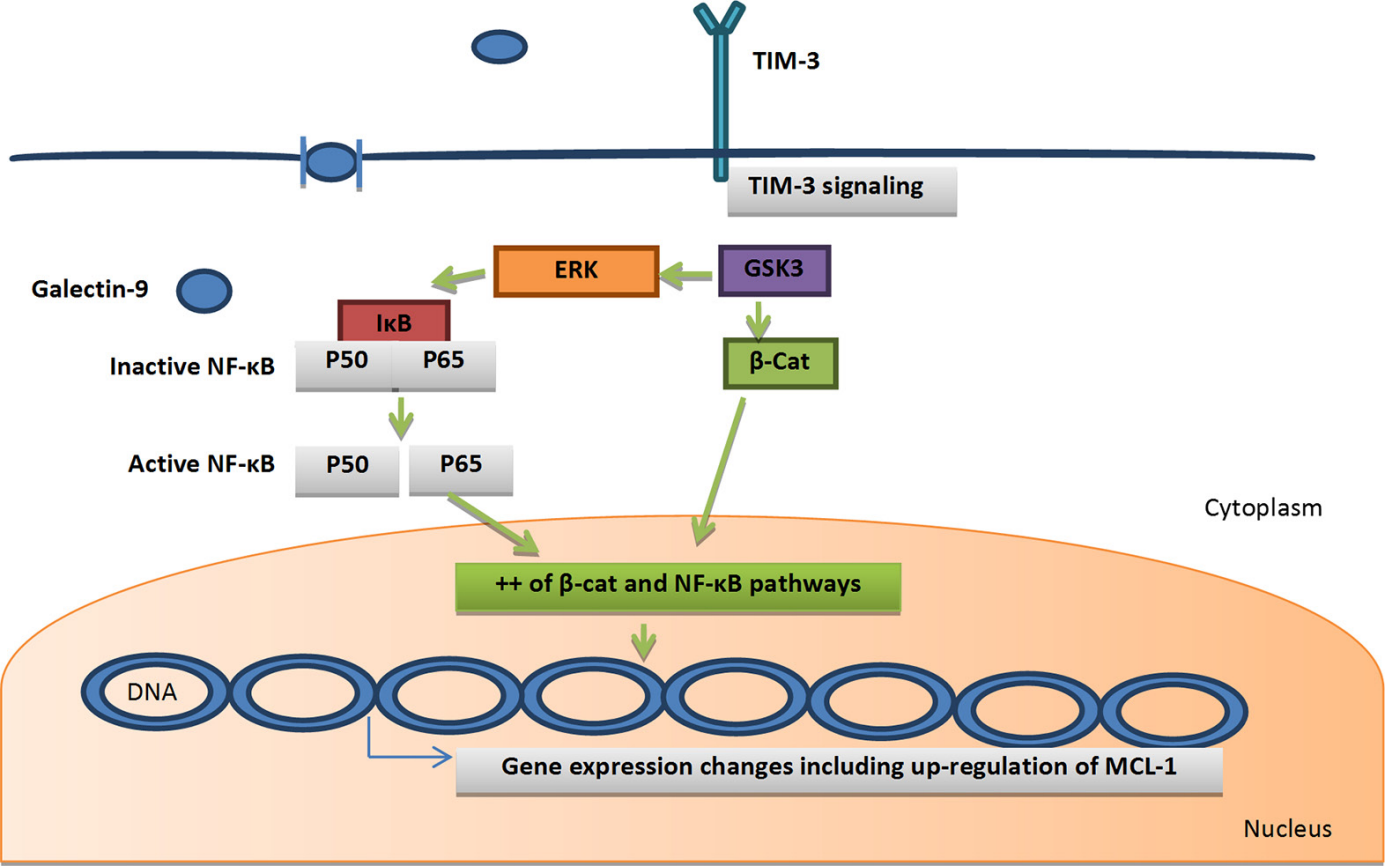
Galectin-9/TIM-3 autocrine mechanism: galectin-9 is mainly produced by TIM-3 +ve leukemic cells Binding of galectin-9 and TIM-3 will induce simultaneous activation of the NF-κB and β-catenin signaling in leukemic cells, which in turn will induce marked gene expression changes including up-regulation of MCL-1, the important survival factor for LSCs, enhancing the pro-survival axis. Green arrows = activation. Abbreviations: β-cat, β-catenin; GSK3, glycogen synthase kinase 3; IκBα, inhibitor of κB; NF-κB, nuclear factor kappa B; MCL-1, Myeloid Cell Leukemia 1.

T cell immunoglobulin mucin-3 (TIM-3) is known as a cell-surface glycoprotein that consists of 4 domains (N-terminal immunoglobulin variable domain, mucin domain, transmembrane domain, and a cytoplasmic tail). The tyrosine residues in the cytoplasmic tail are found in a cluster, suggesting the function of TIM-3 as a signal transduction in AML LSCs. TIM-3 is expressed in the CD34+/CD38− AML LSC fraction and in the majority of their downstream CD38+ leukemic progenitors in most AML types except for M3 [29]. Thus, TIM-3 is suggested to be one of the promising AML LSC surface target molecules [10]. Recent studies demonstrated the strong correlation between TIM-3 and galectin-9, which is increased in AML patients in comparison to the normal individual. Kikushige *et al*. reported that galectin-9 is mainly released by the CD34+ fraction of AML cells that contain LSCs. Binding of galectin-9 and TIM-3 will induce simultaneous activation of the NF-κB and β-catenin signaling in leukemic cells, which in turn will induce marked gene expression changes including up-regulation of MCL-1, the important survival factor for LSCs, enhancing the pro-survival axis (Figure 6). TIM-3/galectin-9 signaling may also be critical for survival of LSCs because it has been found that deprivation of galectin-9 accelerates apoptosis of leukemic cells in AML cell lines [30]. The increased concentration of galectin-9 induced marked gene expression changes including up-regulation of MCL-1. Galectin-9 produced by the LSCs will bind and stimulate TIM-3-expressing AML cells including LSCs (autocrine effect), supporting their survival or leukemia progression [10]. TIM-3 also is originally expressed on the CD4+ Th1 lymphocytes surface and also plays an important role in Th1 cell immunity regulation and tolerance [31].

Kikushige and Miyamoto reported that TIM-3 expression increased in CD34+/CD38- LSCs and blast cells in most AML patients, except for acute promyelocytic leukemia (M3), normal HSCs, and normal progenitors [10]. In testing the inhibitory effect of anti-TIM-3 mAbs on proliferation of human AML in an *in vitro* system, we found that anti-TIM-3 mAbs (0.5 μg/100 μl) was sufficient to interfere with the leukemic cells' proliferation, while the other concentrations didn't show a marked effect on cell survival. These findings also suggest the function of TIM-3 as a signal transduction in AML LSCs. On the other hand, Kikushige and Miyamoto tested the effect of anti-TIM-3 mAb (ATIK2a) *in vivo*. They used NOD/SCID mice for this experiment to test the blocking effect and to potentiate antibody-dependent-cell-cytotoxicity of the mAbs. Inconsistent with our results, injection of anti-TIM-3 mAbs did not affect reconstitution of normal hematopoiesis, but it successfully interfered with the proliferation of the human leukemic cells [10].

### BMI-1

The epigenetic mechanisms are the main player for self-renewal and differentiation of HSCs. One of these mechanisms is under the control of Polycomb-group (PcG) proteins, which are involved in this process by repressing the genes involved in cell proliferation and differentiation [32]. Many previous studies confirmed the strong relation between BMI-1 overexpression and different types of human tumors—breast cancer is one the most powerful examples of this relationship [33, 34]. BMI-1 interacts with several signaling pathways containing Akt, Notch, Wnt, Hedgehog, and receptor tyrosine kinase (RTK). In mammary stem cells, BMI-1 was shown to enhance cell proliferation via its effect of Hedgehog signaling pathway [35].

Many reports discuss the relation between induction of BMI-1 and its effect on the composition of the PcG complex to favor proliferation over cell-cycle arrest, which appeared to be dependent on the relative amounts of BMI-1 in the complex and its biochemical and biologic functions [36]. Besides the important role of BMI-1 in bypassing apoptosis and the controlling of hematopoietic stem cell division, it has recently become clear that BMI-1 also functions in the protection against oxidative stress. In the absence of BMI-1, reactive oxygen species accumulate, activating DNA damage response pathways and enhancing cell apoptosis. The overexpression of BMI-1 will protect both LSCs and blast cells against apoptosis, which may explain the correlation between BMI-1 overexpression and poor prognosis in our patients [37]. Down-modulation of BMI-1 impairs self-renewal and long-term expansion of LSCs and that by turn will be an important strategy in the treatment of AML patients.

Our data showed that *BMI-1* was markedly increased in M1, M5, and M7 groups, while the expression was moderately increased in M2 and M4. The M3 group showed mild increase in *BMI-1* in comparison to the control group and this may be related to the function of BMI-1 as a transcription factor, so that it may be related to increasing the cell division of the bone marrow cells. Iwama *et al.* demonstrated that forced expression of BMI-1 led to a marked expansion of both multi-potential progenitors and HSCs *in vivo* [38]. They also showed that the absence of BMI-1 is related to a profound defect in HSCs' self-renewal [38].

The mechanism of action of small molecule inhibitor PTC-209 is not clear yet, but it may interfere with post-transcriptional regulation of BMI-1 [39]. The higher expression of BMI-1 in the Kasumi cell line compared to KG-1a confirms the role of BMI-1 in cell proliferation in leukemia and the possibility to use BMI-1 as target for new strategies against AML. Kreso and colleagues demonstrated that PTC-209 successfully inhibited the endogenous BMI-1 expression in human colorectal HCT116 and human fibrosarcoma HT1080 tumor cells [39]. The main problem is that BMI-1 is also known to be essential for normal cell survival. So BMI-1 might need to be selectively targeted to the LSCs in order to spare normal cells.

### Limitations

All AML patients were newly diagnosed with no history of myelodysplasia or any therapies (chemo or radiotherapy). Cytogenetic and molecular profiles were not complete for all patients. The number of bone marrow samples from the AML patients was 40, but each group (6 groups) had 3 to 16 patients. We are working on further, larger-scale studies. Another limitation is that TIM-3 is also expressed on T cells, and T cells represent less than 5% of the cells in the normal bone marrow. Blast cells in AML bone marrow samples were more than 90% normalized with healthy bone marrow.

Further studies are required to confirm the expression of these markers in a larger number of patients in correlation to the full chromosomal and molecular studies. We are working towards a target strategy that will attack both extracellular markers (TIM-3 and / or CLL-1) and intracellular molecule (BMI-1) at the same time. Such a strategy might improve the selective targeting of AML blast cells and result in reduced bone marrow cytotoxicity.

## MATERIALS AND METHODS

### Patient and control samples

The AML clinical protocols and the biologic studies were approved by the scientific research and medical ethics committees of Mansoura University, Faculty of Medicine (Mansoura, Egypt). Bone marrow samples of 40 Egyptian patients presenting with CD34+ and CD34- AML were obtained from newly diagnosed AML patients after informed consent in accordance with the Declaration of Helsinki.

Patients had not received any prior treatment for any malignancy and also didn't show any myelodysplasia changes. Diagnosis of patients was based on morphology, cyto-chemistry, immune-phenotyping, and cytogenetics. Our AML groups consisted of M1 (acute myeloblastic leukemia with minimal maturation), M2 (acute myeloblastic leukemia with maturation), M3 (acute promyelocytic leukemia), M4 (acute myelomonocytic leukemia), M5 (acute monocytic leukemia), and M7 (acute megakaryoblastic leukemia). Patient characteristics are shown in Supplementary Data, Table S2. Control normal bone marrow was obtained from 10 patients undergoing splenectomy surgery after informed consent. Patient samples were used for gene expression studies, and cell lines were used for an *in vitro* study of the targeting therapy with MTT assay.

### RNA isolation and qRT-PCR for normal bone marrow and AML patients' bone marrow

For PCR analysis of human bone marrow mononuclear cells (MNCs), the total RNA was extracted using a TRI kit (Sigma) according to the manufacturer's instructions. Complementary DNA (cDNA) was synthesized with the same concentration of RNA in all samples and subjected to qPCR using Realplex Sequence Detection System (Eppendorf, Hauppauge, NY, USA) StepPlusOne thermal cycler (Thermo Fisher Scientific, Waltham, MA) with settings: 95°C for 10 min, followed by 40 cycles of 95°C for 15 s, and 60°C for 1 min. The primers for *CD34*, *TIM-3*, *CLL-1*, *BMI-1*, and *HTRP* primer, house-keeping gene (used as internal control) (Table 1) were obtained from Invitrogen (Grand Island, NY, USA).

**Table 1:** Quantitative RT-PCR primers used (TaqMan gene expression assay)

Gene symbol	Gene description	Assay ID	Amplicon length
CD34	Human CD34 molecule	Hs00990732_m1	91
HAVCR2	Human T cell immunoglobulin mucin-3 (*TIM-3*)	Hs00958618_m1	60
CLEC12A	Human C-type lectin-like molecule-1 (*CLL-1*)	Hs01074333_m1	94
BMI1	Himan B-cell-specific Moloney murine leukemia virus integration site-1 (*BMI-1*)	Hs00995536_m1	76
HPRT1	Human hypoxanthine Phosphoribosyltransferase 1 (House- keeping gene)	Hs02800695_m1	82

All the reactions were performed in triplicate with 20 μl Taqman Universal PCR Master Mix (KAPA Biosystem, Wilmington, MA, USA) containing 1.0 μl cDNA. The relative gene expressions in controls and patients were determined by using the comparative CT (2-ΔΔCt) method.

### Cell lines and reagents

The Kasumi-1 and KG-1a cell lines were from American Type Culture Collection (ATCC, Manassas, VA, USA) and are commonly used as AML cell lines [40, 41].

### Cell death assay (MTT assay)

Cells were seeded in 96-well plates (50 × 10^3^−100 × 10^3^ per well) and were treated with purified monoclonal Ab CD44, CD34 (BD Bioscience), TIM-3, CLL-1 (BioLegend), and BMI-1 small molecule inhibitor (PTC-209) (Xcess Biosciences, San Diego, CA, USA) using 3 different concentrations for each: (4, 2, 1 μg/100 μl) for CD34 and CD44, (1, 0.5, 0.05 μg/100 μl) for TIM-3 and CLL-1, and 4 different concentrations for PTC-209 (0.2, 1. 2, 4 μM). Forty-eight and 72 hour assays were done as described in the Figure legends. The cellular viability of suspended cells was determined with the help of the MTT assay. All the reactions were performed in triplicate. Measured data of cellular viability were normalized using viability values of untreated control cells (100%).

### Statistical analysis

Results are presented as means ± S.E.M. or ± S.D. as indicated in Figure legends. Statistical analysis was performed using one-way ANOVA followed by Newman-Keuls post-test when more than two groups were being compared. Differences between groups were considered statistically significant for *p* ≤ 0.05. All statistical analyses were performed using GraphPad InStat 3 (GraphPad, San Diego, CA, USA). For Kaplan-Meier plot analysis we used GraphPad Prism software and the log-rank test was applied. The log-rank test *p* value indicates the significance of the correlation.

Additional method information is in Supplementary Data.

## CONCLUSIONS

In summary, our data provides indicators about the relationships between the expression level of several LSC-related molecules investigated in this study and the prognosis in our patients. Thus, those known and potentially new markers might be used not only as prognostic markers but also as promising markers for selective targeting of LSCs and for eventually improving the cure rate of AML.

### Supplementary data

Contains experimental methods, flow cytometry figures, and tables of patient characteristics, marker expression in patient groups, and stem cell markers expressed by Kasumi-1 and KG-1a cell lines.

## SUPPLEMENTARY MATERIALS FIGURES AND TABLES


