# Supplementary material for: Acute myeloid leukemia stem cell markers in prognosis and targeted therapy: potential impact of BMI-1, TIM-3 and CLL-1
Source: Oncotarget. 2016 Aug 5;7(36):57811–20. doi: 10.18632/oncotarget.11063 (PMC5295391; doi:10.18632/oncotarget.11063)
Supplement: Supplementary file 1 [file oncotarget-07-57811-s001.pdf]

Acute myeloid leukemia stem cell markers in prognosis and targeted therapy: potential impact of BMI-1, TIM-3 and CLL-1

Supplementary Materials

SUPPLEMENTARY MATERIALS AND METHODS

Isolation of mononuclear cells (MNCs) from bone marrow samples of AML patients and control

Patient and control samples obtained through bone marrow aspirate (5–10 ml) were collected with heparin (anticoagulant). The samples were rapidly prepared using a Ficoll gradient (1.077 g/ml) (Amersham Biosciences, Freiburg, Germany) and subsequent red blood cell lysis. Cells were then frozen in RPMI 1640 with 20% heat inactivated fetal bovine serum (FBS; Sigma, Saint Louis, MO, USA) and 5% DMSO (Riedel-de Haen, Seelze, Germany) in isopropanol-filled containers and subsequently stored in liquid nitrogen. When needed for analysis, cells were thawed and centrifuged to remove the supernatant and pellet was used.

Protein analysis (Western blot technique)

Whole-cell protein extracts were obtained from frozen cell pellets available from 3 patients, 1 control (Hyper-splenism), and 2 AML cell lines (Kasumi-1 and KG-1a), loaded onto a 10% SDS-polyacrylamide gel, and electro-blotted to a PVDF membrane (Bio-Rad). Blocked membranes were incubated sequentially with the monoclonal antibody BMI-1 Antibody (H-99) (Santa Cruz Biotechnology, Dallas, TX, USA), anti-mouse, and detected by enhanced chemiluminescence (Thermo-Scientific, Grand Island, NY) according to the manufacturer's recommendations. Western blot analysis identified a specific band of 37 kDa molecular weight corresponding to a BMI-1 protein.

Flow cytometry analysis

Fresh cells were incubated with monoclonal antibodies for 15 min at room temperature, washed once in PBS containing 0.1% HSA, and analyzed by flow cytometry. Monoclonal antibody combinations contained fluorescein isothiocyanate (FITC)–, phycoerythrin (PE), peridinyl chlorophyllin (PerCP), and allophycocyanin (APC)–labeled monoclonal antibodies.

Anti-CD34 PE, anti-CD38 FITC, anti-CD45 PerCP, Anti-CD44 APC, anti-BMI-1 PE, and Hoechst 33342 were all from BD Biosciences (San Jose, CA, USA). Anti-TIM-3 APC and anti-CLL-1 APC were from BioLegend (San Diego, CA, USA). For the 2 cell lines the main combination was CD34/CD38/CD45/Hoechst33342 - CD34/CD44/CD45/Hoechst 33342 - CD34/CLL-1/CD45/Hoechst 33342 - CD34/TIM-3/CD45/Hoechst 33342.

Data acquisition was performed using a FACS Aria III (BD Biosciences) equipped with an argon and red diode laser, and analysis was performed using Cell Quest software (BD Biosciences). Blasts were identified by CD45dim/low side scatter characteristics according to Lacombe, taking into account that the LSCs' population is a minor population. All analyses were performed in duplicate.

Cell vitality and morphology

Blast cells from both cell lines were assessed every 2 days using Lesheiman`s stain (Sigma) to assess maturation, number, and morphology of myeloblasts. Trypan Blue (Life Technologies, Grand Island, NY) was used to assess the vitality of myeloblasts every 2 days also. Kasumi-1 and KG-1a cell lines showed no differentiation or maturation over 10 passages.

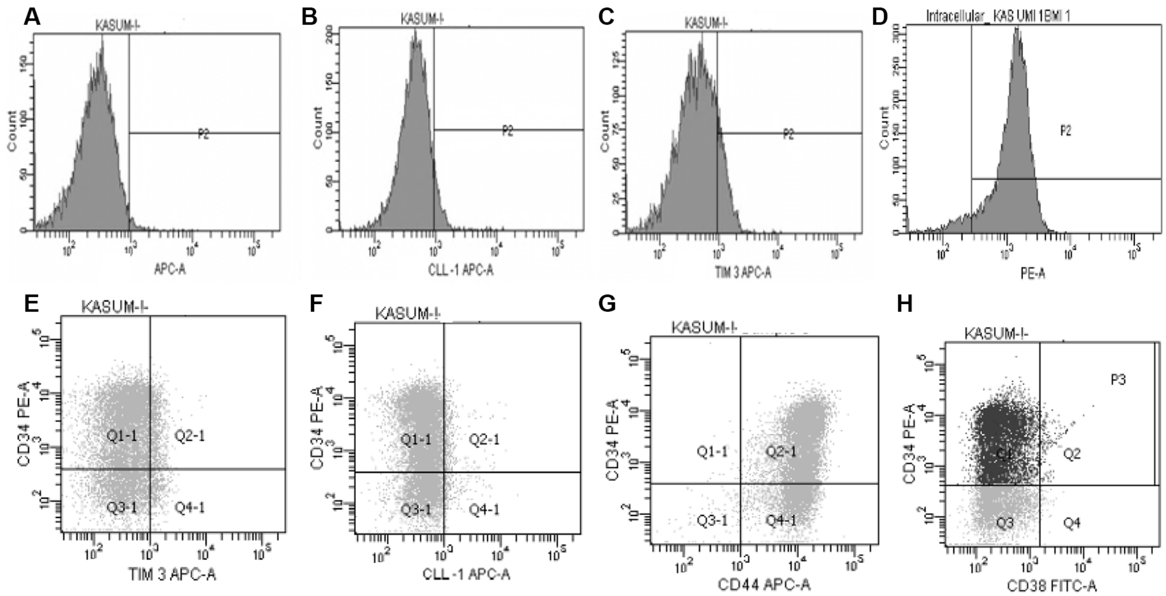

Supplementary Figure S1: Flow cytometric analysis of CD34, CD38, CD44, CLL-1, TIM-3 and BMI-1 expression in Kasumi-1 cell line. (**A**) Isotype control for CLL-1 and TIM-3 expression. (**B**) CLL-1 expression. (**C**) TIM-3 expression. (**D**) BMI-1 expression. **E–H** represent the co-expression of CD34 and the different markers (TIM-3, CLL-1 CD44 and CD38).

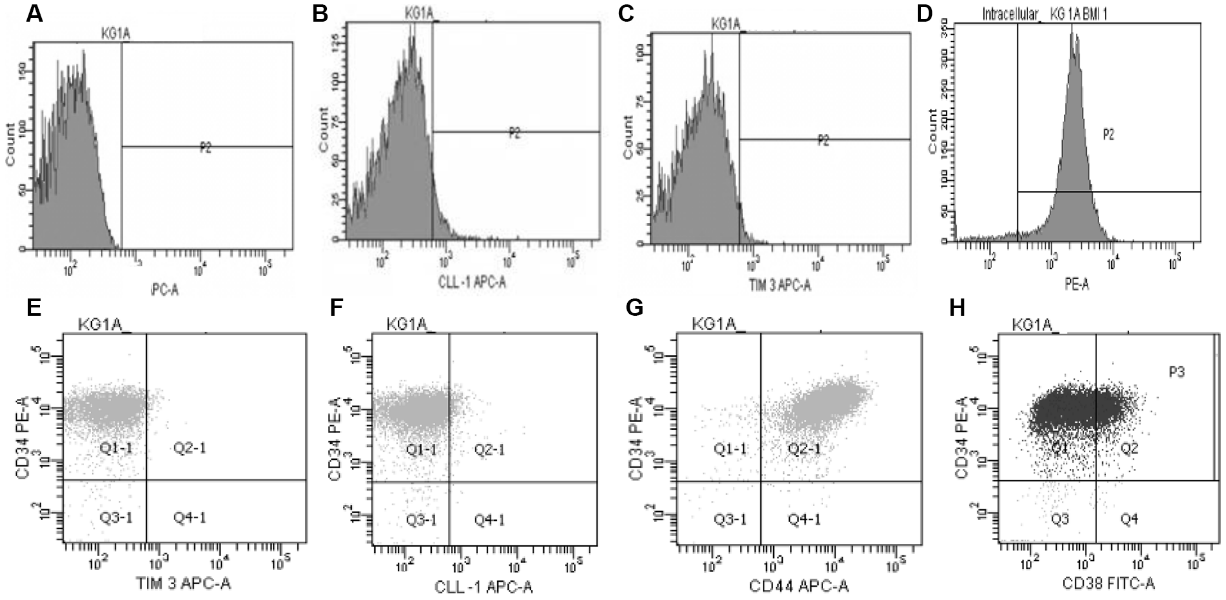

Supplementary Figure S2: Flow cytometric analysis of CD34, CD38, CD44, CLL-1, TIM-3 and BMI-1 expression in KG1-a cell line. (**A**) Isotype control for CLL-1 and TIM-3 expression. (**B**) CLL-1 expression. (**C**) TIM-3 expression. (**D**) BMI-1 expression. **(E–H)** represent the co-expression of CD34 and the different markers (TIM-3, CLL-1 CD44 and CD38).

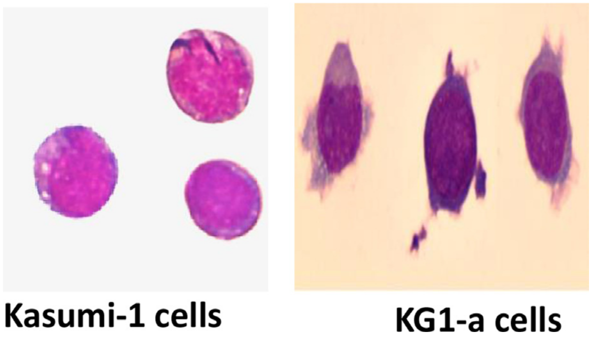

Supplementary Figure S3: Morphology of Kasumi-1 and KG1-a cells under light microscopy (Leishman`s stain). Neither granulocytic nor eosinophilic maturation was observed in the *in vitro* liquid culture up to 10 passages.

Supplementary Table S1: Some stem cell markers expressed by Kasumi-1 and KG1-a cell lines, expressed as percentages, from flow cytometry stem cell marker assay

Cell line	CD34	CD38	CD44	TIM-3	CLL-1	BMI-1
**Kasumi-1**	63	4.1	99	16	8.4	93
**KG-1a**	98	26	98	1.3	4	95

Cell line	CD34/CD38	CD34/CD44	CD34/TIM-3	CD34/CLL-1
**Kasumi-1**	1.6	61	9.3	4.2
**KG-1a**	25	97	1.2	3.7

Supplementary Table S2: Patient characteristics

Patient FAB	No. of patients	No. of males/females	Age	WBCs × 10^6
M1	5	5/0	30–60	6.4–210
M2	7	2/5	31–79	2.3–183
M3	5	3/2	17–62	1.3–92
M4	16	7/9	28–68	2.1–200
M5	4	1/3	17–55	0.3–100
M7	3	1/2	21–28	0.9–14.9
Total	40	19/21	17–79	0.3–210

WBC, white blood cells; FAB, French-American-British classification.
